# Financing of health services for undocumented immigrants in Iran: common challenges and potential solutions

**DOI:** 10.1186/s12992-023-00924-x

**Published:** 2023-04-18

**Authors:** Manal Etemadi, Saeed Shahabi, Kamran Bagheri Lankarani, Seyed Taghi Heydari

**Affiliations:** 1 The National Institute for Health and Care Research Applied Research Collaboration West (NIHR ARC West), University Hospitals Bristol and Weston NHS Foundation Trust, Bristol, UK; 2 Population Health Sciences, Bristol Medical School, University of Bristol, Bristol, UK; 3 Health Policy Research Center, Institute of Health, Shiraz University of Medical Sciences, Shiraz, Iran

**Keywords:** Health Financing, Undocumented Immigrants, Qualitative Study, Iran

## Abstract

**Introduction:**

Iran is host to one of the largest urban refugee populations worldwide, about two million of whom are undocumented immigrants (UIs). UIs are not eligible to enroll in the Iranian health insurance scheme and have to pay out-of-pocket to access most health services. This increases the likelihood that they will delay or defer seeking care, or incur substantial costs if they do seek care, resulting in worse health outcomes. This study aims to improve understanding of the financial barriers that UIs face in utilizing health services and provide policy options to ensure financial protection to enhance progress towards UHC in Iran.

**Methods:**

This qualitative study was conducted in 2022. A triangulation approach, including interviews with key informants and comparing them with other informative sources to find out the complementary findings, was applied to increase data confirmability. Both purposive and snowball sampling approaches were used to select seventeen participants. The data analysis process was done based on the thematic content analysis approach.

**Results:**

The findings were explained under two main themes: the financial challenges in accessing health services and the policy solutions to remove these financial barriers, with 12 subthemes. High out-of-pocket payments, high service prices for UIs, fragmented financial support, limited funding capacity, not freeing all PHC services, fear of deportation, and delayed referral are some of the barriers that UIs face in accessing health care. UIs can get insurance coverage by using innovative ways to get money, like peer financing and regional health insurance, and by using tools that make it easier, like monthly premiums without policies that cover the whole family.

**Conclusion:**

The formation of a health insurance program for UIs in the current Iranian health insurance mechanism can significantly reduce management costs and, at the same time, facilitate risk pooling. Strengthening the governance of health care financing for UIs in the form of network governance may accelerate the inclusion of UIs in the UHC agenda in Iran. Specifically, it is necessary to enhance the role of developed and rich regional and international countries in financing health services for UIs.

## Introduction

The number of refugees and undocumented immigrants (UIs), who are outside the regulatory norms of the country they are in [1], has steadily increased over the past several decades [2]. Due to climate change, it is predicted that there will be between 140 and 200 million UIs worldwide by 2050 [3, 4]. Physical and mental healthcare needs for refugees and UIs can be substantial [5–7], yet their socioeconomic status is often lower, and their employment status may limit their care seeking [8]. At the moment, most of the cost of health services for refugees and UIs falls on host countries. This can be hard for low- and middle-income countries (LMICs) that are trying to get universal health coverage (UHC) for their own populations. This is true even though developing countries host nearly 90% of the world’s refugees [9]. Middle-income countries are especially disadvantaged by their ineligibility for certain types of international financing [10]. To achieve UHC, it is essential that effective and sustainable health financing arrangements exist to cover refugees and UI [8, 11]. Access to affordable health care is also key to global health security and containing outbreaks, such as COVID-19 [10]. The Global Compact for Refugees recognizes that the burden on host countries needs to be lessened [12] and looks into the possibility of new, creative, and more reliable ways to pay for health care [13].

The Islamic Republic of Iran is host to one of the largest urban refugee populations worldwide. While a sizable number of Afghan migrants had been present in Iran since the late 19th century, Afghans began to arrive in large numbers in Iran after the Soviet army occupied Afghanistan in 1979. Other displacement movements from Afghanistan into Iran occurred after the emergence of the Islamic State group in Afghanistan in 2014. Around 2.1 million undocumented Afghans were living in Iran as of 2020, while estimates from the second and third quarters of 2022 put this number at 2.6 million. Following the Taliban takeover of Afghanistan in August 2021, many Afghans fled to Iran due to the deterioration of the security and humanitarian situation in their country. As of September 2022, the Iranian government had recorded approximately one million new Afghan arrivals.

Almost 96% of Afghan refugees live in urban areas, while the other 4% live in approximately 20 refugee settlements across the country. 55% of Afghans living in Iran (both registered refugees and undocumented immigrants) lived in Tehran, Isfahan, and Razavi Khorasan [14], which are some of the richest provinces in Iran and have very good health care systems [15]. Of those registered by UNHCR as newly arrived as of early 2022, 45% were under the age of 18, and 58% were female. 23% of households were female-headed [16].

The situation for undocumented Afghans in Iran is not clear [17]. Afghan patients who are illegal may be treated, but no records are kept for them. Being undocumented is statistically associated with substance use, as Afghan households had at least one adult member using illegal drugs (4.2% out of 2,034 Afghans). Moreover, food insecurity was significantly more prevalent among female-headed households, but also in those families without legal residential status [18].

UIs represent the largest number of Afghans in Iran, even though their exact numbers are lacking. They do not have an official residence permit, and thus no official access to health care. However, they are rarely refused health services. Some seem to also have access to complex services such as hemodialysis or kidney transplantation. Nevertheless, because of their lack of insurance and status as undocumented migrants, Afghan women do not receive free prenatal care, as do Iranian women. Although most Afghan migrants speak Persian, the official language of Iran, some differences in accents and a low literacy rate among migrants can function as barriers to health education programs [19, 20]. Cultural belief stops the refugees to refer to the health centers and care about the health issues being particularly observed in the undocumented refugees [21].

The Iranian health system is predominantly public, and services such as emergency care, routine vaccination, and health care for communicable diseases are free, and there is good access to primary health care for undocumented migrants [22]. All primary healthcare is free of charge, regardless of documentation status. Hospital care is also available, however, at a higher rate for them than for nationals [23]. Moreover, the Iranian health system provides HIV services to documented and undocumented immigrants. This issue was identified as a facilitating factor for improving access to such services for Afghan immigrants at the policymaking level [24]. However, a study mentioned that UIs don’t have the right to be admitted to public hospitals, even if they are in an emergency situation. Doctors face difficulties in these situations because they are unable to refer patients for further treatment [25]. The high cost of some secondary and tertiary prenatal services, such as screening tests and prenatal sonography, reduces utilization of such services by Afghan women, particularly undocumented women, and puts them at a higher risk for adverse perinatal outcomes because they usually visit private facilities in fear of being arrested and deported at government facilities [17].

In 2015, Iran allowed documented refugees the possibility of enrolling in the national health insurance scheme to benefit from a comprehensive package of health care interventions at public and private providers, similar to that available to Iranians. The United Nations High Commissioner for Refugees (UNHCR) currently finances the premium for vulnerable refugees (about 12% of the total). However, as UNHCR funding is increasingly constrained, it’s unclear how it will continue to respond to rising needs. The remaining registered refugee population has to pay the premium out of pocket. Many refugees are not able to afford the premium costs and can no longer cover their most basic needs, let alone the cost of health insurance, which is estimated to represent some 40% of an average refugee family’s monthly expenditure [26].

In contrast, UIs are not eligible to enroll in the health insurance scheme. While they, like all Iranians, can access free primary preventive care services [27], they have to pay out of pocket for curative or specialized hospital services. This increases the likelihood that they will delay or defer seeking care, or incur substantial costs if they do seek care, resulting in worse health outcomes [17]. Together, the economic crisis in Iran and the social and economic effects of COVID-19 are making this problem worse and making it harder for people to get the care they need [28].

While previous studies have examined the financial costs of care seeking for UIs, there has not to date been a comprehensive assessment of the financial access barriers facing UIs and the potential policy options to improve financial protection for UIs in Iran. There are potentially valuable lessons that can be drawn for Iran from other middle- and high-income countries. The goal of this study is to learn more about the financial barriers that make it hard for UIs to get care and to offer different policy options for protecting UIs’ finances and making more progress toward UHC in Iran.

## Methods

### Study design

This qualitative study was conducted from January 2022 to July 2022 based on the Standards for Reporting Qualitative Research (SRQR) criteria [29] and the Critical Appraisal Skills Programme (CASP) Qualitative Checklist [30]. A triangulation approach, including interviews with key informants and comparisons with other informative sources to find out the complementary findings, was applied to increase data confirmability. Complementary data were collected through document analysis to ensure methodological triangulation. The Institutional Review Board (IRB) of Shiraz University of Medical Sciences looked over the plan for this study and gave its approval (No. 26,061) after making the changes that were needed.

### Sampling and recruitment strategy

Both purposive and snowball sampling approaches were used to select participants. First, the research team prepared a list of potential samples and tried to communicate with them according to their previous acquaintance with the stakeholders involved in financing and providing health services needed by refugees and asylum seekers in Iran. In the next step, in order to ensure maximum diversity, the participants were asked to introduce knowledgeable people who could provide information in this field. Sampling continued until data saturation was reached and no new data was provided. After getting the initial consent of the people to conduct the interview, a consent form containing general information about the research was sent to them via email or WhatsApp. In this form, it was also guaranteed that the participants’ information would remain completely confidential, and they were free to withdraw freely at any stage of the study.

In the second phase, to retrieve the relevant documents, we searched the official websites of the Ministry of Interior, the Islamic Studies Center of Majlis [Parliament], the Ministry of Health and Medical Education, the Ministry of Labor and Social Affairs, and the Iranian Red Crescent Society. The search key terms were “refugees,“ “immigrants,“ “health,“ “law,“ “legislation,“ and “regulation.“

### Data collection

Individual face-to-face, semi-structured interviews were conducted by M.E. (a female health policy Ph.D. graduate with a scientific and executive background in the field of health financing) in a quiet room in participants’ workplaces. Before each interview, the interviewer explained the research’s goals and how it would be done. If the person being interviewed agreed, the meeting could begin. In order to facilitate the interview process, an interview guide containing open questions was used, which were mainly based on the UHC cube (Table 1). Based on the feedback received from the initial interviews, the questions were revised for greater clarity. In addition to the audio recording of the interview sessions, notes were also taken by the interviewer (M.E.) to be used later in the data analysis process. After the end of each interview, the recorded file was transcribed verbatim and saved anonymously in the word processing software.

**Table 1:** Interview guide

**Open-ended questions**
1. What are the existing challenges of health financing for immigrants in Iran? *Probes: What is your own experience in facing Undocumented Immigrants (UIs) struggle to access health services?*
2. What are the barriers to financial access to health services for UIs in Iran?
3. What should be the role of the government and international institutions in health financing for UIs?
4. Which policies and programs have been adopted so far to improve the financing of health services for UIs? What have been their results in terms of improving financial access?
5. What is the role of domestic and international non-governmental organizations in financing the health of UIs?
6. In what way can you seek more international support for financing the health of UIs?
7. What are your potential solutions to improve the financing of health services for undocumented immigrants? *Probes: what about governance of financing? What about revenue collection, resource pooling, and purchasing of health services?*

In respect to the document analysis step, a researcher-made data collection form was used. The validity of the form was evaluated and confirmed by the research team and five experts using the content validity assessment method. The documents were analyzed regarding the protection policies and legislation for refugees and immigrants in the case of health services.

### Data analysis

The data analysis process was done in parallel with the data collection and was based on the thematic content analysis [31]. Firstly, two researchers (M.E. and S.SH.) frequently reviewed the texts in order to familiarize themselves with the collected data and determine the meaning units. After that, the codes were assigned meanings in units. Any disagreement at this stage was resolved through discussion and, if necessary, through the participation of a third expert researcher (K.B.L.). After reaching an agreement on the obtained codes, they were categorized and assigned to the two main themes, including challenges and policy solutions. The process of data analysis was mainly done through MAXQDA 11 software (VERBI GmbH, Berlin, Germany), but in some cases, manual analysis was also done. Several researchers with different executive and scientific backgrounds worked on the data analysis process to make it stronger and better.

### Rigor and trustworthiness

In qualitative studies, different approaches are considered in order to improve the credibility, confirmability, authenticity, transferability, and dependability of the findings [32]. In the present study, the first author was immersed in the study for a long time, and relevant experts were used to check the credibility of the data analysis process. In addition, the written texts and the data analysis results were provided to participants to review and approve the findings (confirmability). The research team attempted to include direct codes from nearly all participants to ensure authenticity. Furthermore, as mentioned earlier, the highest diversity was adapted during sampling to enhance the transferability of findings. In the end, data analysis was conducted, involving researchers with different backgrounds (dependability).

## Results

### Findings from the document analysis phase

**Fig. 1:**
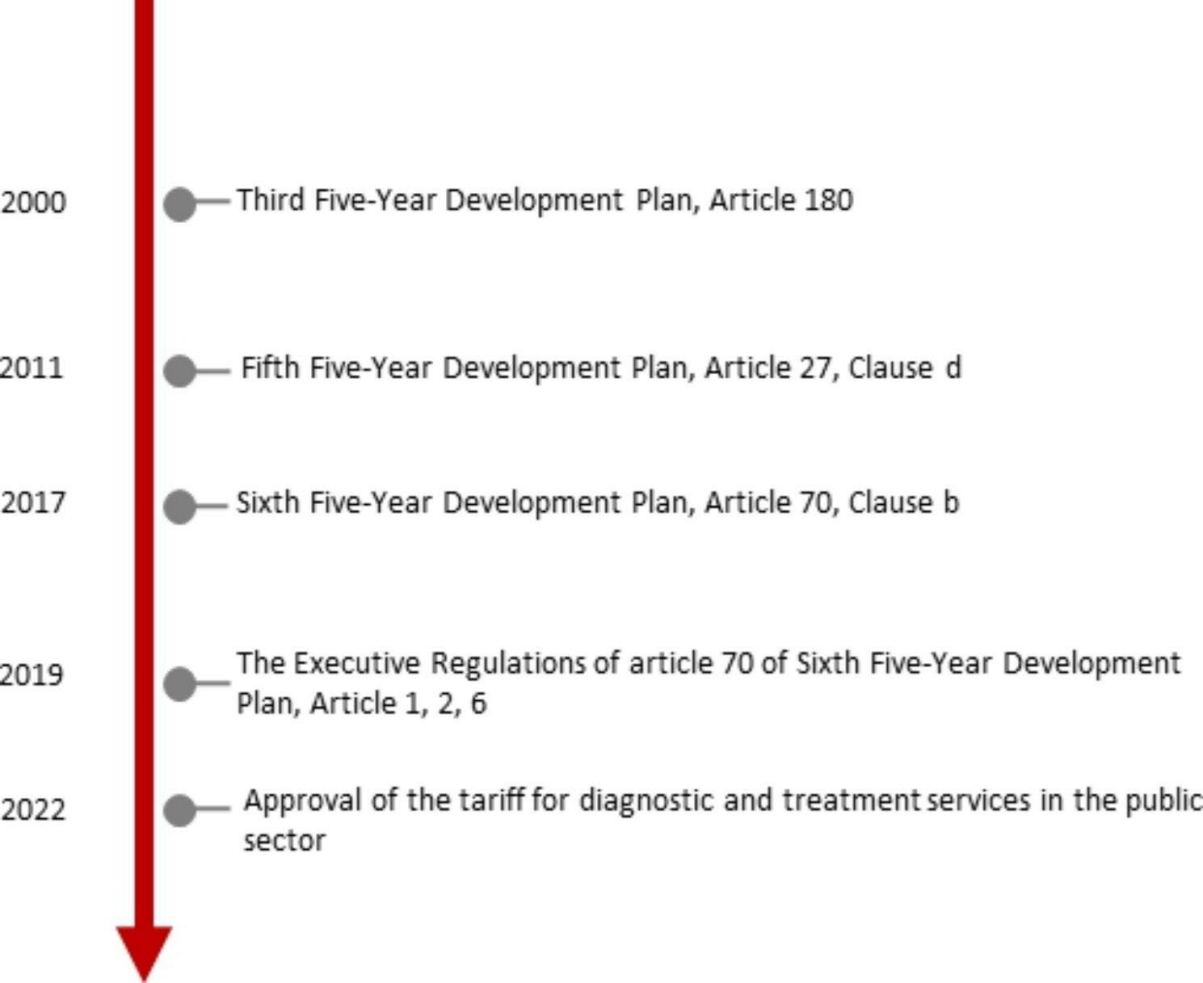
History of relevant key policy documents

Although initially 10 potential documents related to financing and providing services to immigrants were found, after the review, only five documents were considered for analysis in this study (Fig. 1).

Based on the findings, official refugees, or those with a valid Amayesh card (for Afghan immigrants), or Identity card (for Iraqi immigrants) are eligible for basic health insurance coverage in Iran based on a tripartite agreement (the Ministry of the Interior, the High Commissioner for Refugees, and the Health Insurance Organization). Until 2019, 100% of the insurance premiums of vulnerable refugees, including people with special and incurable diseases or people with low income, were paid by the UNHCR. Except for vulnerable groups, others should pay 100% of their insurance premiums and those of their dependent families.

According to the Sixth Five-Year Development Plan, insurance premiums for vulnerable beneficiaries must be paid for 50% (50%) by the government and 50% (50%) by individuals, or, in the case of realization, from the location of international aid for refugees. The health insurance organization must propose the credits needed to implement this law annually, and the Program and Budget Organization of the country must include it in the annual budget bills.

These policies have increased vulnerable refugees’ access to health care and reduced financial barriers for them, but the cost of insurance premiums and cost sharing are still an obstacle for other refugees and have had limited effects on financial protection. Also, despite the permission of the law to provide health insurance coverage to all foreign nationals residing in the country in accordance with the Sixth Five-Year Development Plan, it is not possible to enroll in the health insurance system for UIs in Iran, and they face financial obstacles to accessing the services, so they have to pay for the services out-of-pocket (OOP).

### Findings from the qualitative interview

As a whole, 17 individuals from IHIO, UNHCR, the Ministry of Interior, the Ministry of Health, and the Iranian Red Crescent Society agreed to participate in individual interviews. The findings of the study are explained under two main themes: the financial challenges the UIs faced in accessing health services in Iran and the policy recommendation to remove these financial barriers (Table 2).

**Table 2:** Summary of themes and sub-themes

Themes	Sub-themes		Quotes
Challenges	High out-of-pocket payment for UIs		*“Our problem is with people who are in the country illegally. They naturally do not receive [health] services because they have to pay OOP when they visit.“P6 (Male/ MOHME/14 years experiences)*
	High medical tariffs for UIs		*“For immigrants without identity documents, we do not emphasize and define the Iranian tariff for them. The approved annual tariff is specific to Iranian nationals and we have their own circular for non-Iranian nationals.“P7(Female/ MOHME/15 years experiences)*
	Fear of deportation		*“For instance, a diabetic illegal immigrant who could be treated with a low-cost medication in the beginning avoids going to medical facilities out of fear of being detained…” P8 (Male/ IHIO/23 years experiences)*
	Patient escape phenomenon		*“Some of them [UIs] may escape. Because the cost of some treatments is very high. And that patient also had no way but escape.“ P6 (Male/ MOHME/14 years experiences)*
	Delayed referral		*“In comparison to those who have an allowance and are in some way legal, those [immigrants] who are here illegally are far weaker and more vulnerable.“ P5(Male/ IHIO/20 years experiences)*
	Fragmented financial support		*“For instance, if an undocumented Afghan worker falls down during a construction accident, breaks his arm and leg, or has his spinal cord severed, and needs to be taken to the hospital, the cost to the health system will be close to 5 billion Rials…"P15 (Male/ MOHME/15 years experiences)*
	Limited funding capacity		*“With the sanctions and financial challenges, we encounter in our country, even the international fund which enter to the country is even not the rate we expected based on the refuges population in our country.” P4 (Male/ Ministry of Interior/20 years experiences)*
Policy solutions	Creating an insurance coverage for UIs		*“It should be mandatory for them to be insured, that is, if they are healthy, we should take money from them for their unhealthy ones.“ P8 (Male/ IHIO/23 years experiences)*
	New revenue sources for insurance coverage of UIs	Using the capacity of Afghans with adequate financial resources to support the UIs	*“The Afghans who are in Iran and have very good financial resources should create an assembly like the donor assembly, a fund to help their poor countrymen…"P6 (Male/ MOHME/14 years experiences)*
		Earmarking a proportion of taxes paid by immigrants	*“There are various taxes that this population pays for staying in the country. A part of it can be given to the health system to help with the cost of their medicine and treatment. It can be a good source of funding.“P7 (Female/ MOHME/15 years experiences)*
		Receiving monthly insurance premiums from immigrants	*“The insurance companies can take the insurance premiums monthly. There is also an electronic system that renews them monthly. In this case, although I believe you should not work in such a way that it is an adverse selection that the sick will just come"P7(Female/ MOHME/15 years experiences)*
		Using the resources of international organizations such as the UNHCR	*“When there are 300,000 to 350,000 UIs in a province, the UNHCR should provide a global amount as income to the Ministry of Health or health insurance so that the UIs can go to certain medical centers and be treated.“ P12 (Male/ IHIO/21 years experiences)*
		Considering lower premiums for UIs	*“The health insurance premium for these [refugees] is about 80% higher than that of Iranians. The government should lower the insurance premium rate to encourage people to get insurance"P6 (Male/ MOHME/14 years experiences)*
	Accurate registering of information		*“The Ministry of Interior has started the process of identifying illegal and unregistered immigrants because if you want to work in the insurance field, you must first know their identity"P16 (Female/ IHIO/11 years experiences)*
	Strategic purchasing		*“It is possible to create a series of special clinics for immigrants in different parts of big cities. With this method, immigrants will know where to go to receive health services.“P8 (Male/ IHIO/23 years experiences)*
	Strengthening the governance	Taking advantage of the capacity of international and regional organizations	*“International organizations such as the United Nations have huge financial resources that should be allocated to countries such as Iran that are facing limited resources in order to provide health services to vulnerable migrants…” P17(Male/ Medical University/6 years experiences)*
		Developing a national health map for UIs	*“It is not a clear system in terms of implementation. In my opinion, the description of the national duties in this regard has not yet been formulated, which means we do not know which institution is in charge at the national level!?“ P10 (Male/ IHIO/17 years experiences)*
		Moving towards ex-ante financing approaches	*“Now, most of the time, when the migrant crisis worsens, resources are allocated to finance their health services. While there is a need to allocate their financial resources before a crisis occurs, in addition to better risk distribution, it also provides a platform for more appropriate accumulation of collected financial resources.“ P17 (Male/ Medical University/6 years experiences)*
		Establishing a joint headquarters and participatory governance	*“The government must form a council for this issue. The head of the council must be the first vice president so that they can move forward, because 5 million people is not a small number. If we want to think about it inter-sectoral, I think it will be simple and quick. These should be looked at comprehensively and intra sectoral"P11 (Male/ IHIO/20 years experiences)*

### Challenges

#### High out-of-pocket payment for UIs

UIs in Iran do not have financial access to health services and have to pay the entire cost OOP. Many of them do not follow up on their health problems and do not go to medical centers for fear of the cost of treatment.

*“Our problem is with people who are in the country illegally. They naturally do not receive [health] services because they have to pay OOP when they visit.“ P6 (Male/MOHME/14 years of experience)*.

#### High medical tariffs for UIs

Due to the fact that UIs don’t have insurance, the cost of treating them is not set. However, it is usually much higher than the normal tariff set by the Council of Ministers, and the hospital can charge them up to several times the formal tariff.

*“For immigrants without identity documents, we do not emphasize and define the Iranian tariff for them. The approved annual tariff is specific to Iranian nationals, and we have their own circular for non-Iranian nationals.“ P7 (Female/MOHME/15 years of experience)*.

Primary health care services are provided free of charge to all people living in the country, whether Iranians or immigrants, in comprehensive health centers. Nonetheless, they should pay out-of-pocket prices for medical services in these centers.

*“The Ministry of Health and Medical Education provides free primary care to all resident refugees as well as Iranians. In fact, only preventive services are free for this group of people, and they [UIs] have to pay out of pocket to receive medical services.“ P4 (Male/Ministry of Interior/20 years of experience)*.

#### Fear of deportation

In fact, many of these immigrants don’t go to medical centers because they are afraid of being deported out of the country and sent back to their home country, which has political problems.

*“For instance, a diabetic illegal immigrant who could be treated with a low-cost medication in the beginning avoids going to medical facilities out of fear of being detained. However, when his condition worsens to the point where amputation is necessary, he visits a hospital. Situations that put our health system under a lot of financial strain.“ P8 (Male/IHIO/23 years of experience)*.

#### Patient escape phenomenon

During the interviews, the people who took part talked about how not being able to pay for treatment costs in UIs causes patients to run away, which is expensive for the health system.

*“Some of them [UIs] may escape. Because the cost of some treatments is very high. And that patient also had no way but to escape.“ P6 (Male/MOHME/14 years experiences)*.

#### Delayed referral

Due to lateness and non-referral, the health status of UIs is typically more problematic. They refer for services when the disease is in its acute phase and they have comorbidities. The patient and the healthcare system would both have to pay more if something similar occurred.

*“In comparison to those who have an allowance and are in some way legal, those [immigrants] who are here illegally are far weaker and more vulnerable.“ P5 (Male/IHIO/20 years of experience)*.

#### Fragmented financial support

Despite the lack of clear laws and maps in the field of UIs in the country, these people are subject to the support of the hospital’s social work unit if they go to public hospitals. In the instruction of economic support for poor patients in public hospitals, the inclusion of economic support for all people who refer to medical centers, including Iranians, immigrants, and unknown people, is foreseen, and therefore, in the case of hospitalization and an inability to pay, is supported by the social work unit.

*“For instance, if an undocumented Afghan worker falls down during a construction accident, breaks his arm and leg, or has his spinal cord severed, and needs to be taken to the hospital, the cost to the health system will be close to 5 billion Rials. These folks are being served and are not being rejected, as evidenced by the fact that this person has spent 18 months in the hospital and has had three wound procedures.“ P15 (Male/MOHME/15 years of experience)*.

Some vulnerable UIs with rare and incurable diseases are covered by the insurance system on a case-by-case and special basis through the Ministry of Interior, and they are financially protected.

*“Some folks occasionally experience serious issues. For instance, they have cancer, are receiving dialysis care, or have acute issues. They receive assistance from the Immigration Department (ministry of interior) itself, which issues temporary travel documents and introduces them to health insurance. The truly vulnerable are covered by health insurance.“ P4 (Male/Ministry of Interior/20 years of experience)*.

The Volunteer Organization of the Red Crescent Society has a cooperation with the social work unit of public hospitals, which, in the case of poor patients, whether Iranian or non-Iranian, accepts financial aid to reimburse hospitals in the public sector.

*“Unofficial immigrants may have something happen to them and be hospitalized in public hospitals. If the public hospital issues an official invoice, we (Red Crescent Volunteer Organization) will help.“ P14 (Male/Red Crescent Society/18 years of experience)*.

Furthermore, organizational or individual charities that have a direct relationship with the social work unit of hospitals or operate as institutions independent from the hospitals mainly provide financial support to non-Iranian immigrants as well.

*“There are charities in Iran that, in addition to doing good things for Iranians, are also doing work for foreign immigrants. They play a very important role.“ P14 (Male/Red Crescent Society/18 years of experience)*.

#### Limited funding capacity in Iran

Iran faced a financial crisis due to economic sanctions, and this made health financing a challenge for the government even for Iranians. When it comes to refugees and immigrants, it is even more challenging. While Iran is experiencing economic hardship, it has committed to paying half of the premium for the refugees’ health insurance, and there is no additional capacity to spend on UIs.

*“With the sanctions and financial challenges we encounter in our country, even the international funds that enter the country are not at the rate we expected based on the refugee population in our country.” P4 (Male/Ministry of Interior/20 years of experience)*.

### Policy solutions for financial protection to UIs

#### Creating a separate insurance fund for UIs

Since Iran’s general health policies say that the insurance system is the main way to pay for health care, every way for immigrants to pay for health care should be worked out through the insurance system and through mandatory policies.

*“It should be mandatory for them to be insured; that is, if they are healthy, we should take money from them for their unhealthy ones.“ P8 (Male/IHIO/23 years of experience)*.

This insurance coverage can be defined in various formats, including temporary insurance for people who stay in the country temporarily and for a short period of time, and permanent insurance for people with a longer stay, for which the premium will be defined differently in each case.

*“The best solution is insurance. It can be temporary insurance with a higher cost, or it can be for those who are official and permanent, like the Iranians. Because you don’t have anything called “risk pooling,“ you have to pay more money. But the one that comes stays as long as it is, and risk pooling and risk distribution happen.“ P6 (Male/MOHME/14 years of experience)*.

### New revenue sources for insurance coverage of UIs

#### Using the capacity of Afghans with adequate financial resources to support the UIs

The individuals who took part thought that many people with money and the same race as immigrants who live in Iran have the ability and desire to help and protect the health of immigrants, and that we can use this potentially valuable resource by getting rid of the political barriers that are in the way.

*“The Afghans who are in Iran and have very good financial resources should create an assembly like the donor assembly, a fund to help their poor countrymen. In fact, a separate fund for immigrants can be created, and these financial resources can be used to strengthen it. We have businessmen from Afghanistan who live in Europe or America and are willing to fully cover the cost of health insurance for immigrants.“ P6 (Male/MOHME/14 years of experience)*.

#### Earmarking a proportion of taxes paid by immigrants

Immigrants, like Iranians, are subject to value-added tax and fees for using some services. The incidence and prevalence of diseases in this population are high, so the health system needs to attract resources to finance the provision of health services to them. Therefore, it is necessary to allocate part of these financial resources to the health system in order to provide health services to this group.

*“There are various taxes that this population pays for staying in the country. A portion of it can be given to the health system to help with the cost of their medicine and treatment. It can be a good source of funding.“ P7 (Female/MOHME/15 years of experience)*.

#### Receiving monthly insurance premiums from immigrants

The discussion of dividing insurance premiums and the possibility of paying them monthly to help immigrants, such as the administrative system where insurance premiums are deducted from employees’ salaries on a monthly basis, can be a helpful option to increase the ability of immigrants to pay for their insurance premiums.

*“The insurance companies can take the insurance premiums monthly. There is also an electronic system that renews them monthly. In this case, although I believe you should not work in such a way that it is an adverse selection, the sick will just come” P7 (Female/MOHME/15 years of experience)*.

#### Using the resources of international organizations such as the UNHCR

UNHCR does not consider itself responsible for UIs as they are not classified as refugees and do not fall within the UN definition of legal protection. However, considering the conditions of the country of origin of the immigrants and making the international community, especially the World Health Organization, more sensitive to the health of the UIs living in the country, the resources of this agency and other UN agencies can be used to help these groups. This money can be given to the insurance system or universities in the form of a budget for the hospitals to use as they see fit.

*“When there are 300,000 to 350,000 UIs in a province, the UNHCR should provide a global amount as income to the Ministry of Health or health insurance so that the UIs can go to certain medical centers and be treated.“ P12 (Male/IHIO/21 years of experience)*.

#### Considering lower premiums for UIs

Some of these individuals have the financial ability to contribute toward the cost of insurance premiums. The likelihood of covering these people will be improved by lowering the insurance premium and bringing it down to the per capita level of Iranians.

*“The health insurance premium for these [refugees] is about 80% higher than that of Iranians. The government should lower the insurance premium rate to encourage people to get insurance.“ P6 (Male/MOHME/14 years of experience)*.

According to the experience of non-vulnerable refugees in the country during the last decade, due to the high insurance premiums and the high size of the household, and the whole payment of insurance premiums at the point of coverage registration from their pocket, they often do not take insurance coverage and turn to the phenomenon of fraud in the case of using others’ insurance. The same experience will be repeated with UIs. As a result, eliminating the requirement that their size determine their ability to pay the insurance premium will assist this group of immigrants in paying the premium.

*“If the insurance institutions remove the condition of the number of family members for calculating the insurance premium, they will certainly welcome it…” P2 (Male/UNHCR/15 years of experience)*.

On the other hand, it is possible to make an insurance plan with a lower premium and a smaller service package that includes only the most important services and fits the health needs of immigrants.

*“We can define a smaller service package according to their needs, and at least if the insurance coverage is to be based on the principles of cost-effectiveness calculation. Indeed, we have to create a separate service package specifically for them according to their situation.“ P10 (Male/IHIO/17 years of experience)*.

In fact, since it is not possible for us to obtain insurance premiums according to their income status because statistics on their assets and income are not recorded, the means test for estimating the insurance premium rate is not applicable for them.

### Accurate registering of information

One of the main prerequisites for financing the health services needed by immigrants in Iran is the accurate registration of their information. In this way, Iran’s Ministry of Interior has asked that information about UIs be registered. This is a step toward making an insurance system for them.

*“The Ministry of Interior has started the process of identifying illegal and unregistered immigrants because if you want to work in the insurance field, you must first know their identity.“ P16 (Female/IHIO/11 years of experience)*.

### Strategic purchasing

Based on the questions that were asked during the interviews, one of the best ways for UIs to buy health services strategically is to buy based on the organized system of providing health services, which includes the family doctor, the referral system, and special centers for their referral.

*“It is possible to create a series of special clinics for immigrants in different parts of big cities. With this method, immigrants will know where to go to receive health services.“ P8 (Male/IHIO/23 years of experience)*.

### Strengthening the governance

#### Taking advantage of the capacity of international and regional organizations

The need to effectively use the capacities of international and regional organizations was one of the suggested solutions in many of the interview sessions. In fact, the people who took part thought that, since Iran is one of the countries that takes in the most immigrants in the region and the world, it is necessary to set up joint financial funds on a global and regional level in order to give health services to vulnerable groups of immigrants.

*“International organizations such as the United Nations have huge financial resources that should be allocated to countries such as Iran that are facing limited resources in order to provide health services to vulnerable migrants. Also, there are very wealthy countries in the Middle East region that can be helped to finance the health services needed by immigrants by forming regional financial funds.“ P17 (Male/Medical University/6 years of experience)*.

#### Developing a national health map for UIs

In the field of health for UIs, many institutions at the level of the ministries and national and international organizations participate, but specifically, no institution has been recognized as a steward for health financing and insurance coverage for this group, and the tasks and duties of each institution are not clearly defined.

*“It is not a clear system in terms of implementation. In my opinion, the description of the national duties in this regard has not yet been formulated, which means we do not know which institution is in charge at the national level!“ P10 (Male/IHIO/17 years of experience)*.

#### Moving towards ex-ante financing approaches

According to the findings, a portion of participants believe that an ex-ante approach to financing health care services for UIs is required. In fact, they argue that adapting such a strategy can not only facilitate risk distribution and effective pooling of revenues but also lead to more transparency and fewer corrupt activities.

*“Now, most of the time, when the migrant crisis worsens, resources are allocated to finance their health services. While there is a need to allocate their financial resources before a crisis occurs, in addition to better risk distribution, it also provides a platform for more appropriate accumulation of collected financial resources.“ P17 (Male/Medical University/6 years of experience)*.

#### Establishing a joint headquarters and participatory governance

The issue of the security and health of UIs is a cross-departmental issue and not within the scope of performance, authority, and responsibility of one ministry alone. A multi-sectoral council should be formed with the agenda of improving the health of immigrants to determine the duties of each institution and hold them accountable for the assigned tasks.

*“The government must form a council for this issue. The head of the council must be the first vice president so that they can move forward, because 5 million people is not a small number. If we want to think about it inter-sectorally, I think it will be simple and quick. These should be looked at comprehensively and intra-sectoral” P11 (Male/IHIO/20 years of experience)*.

## Discussion

According to the findings of this study, the provision and financing of health services for UIs in Iran is associated with many challenges, including high out-of-pocket payments, high service prices for UIs, fragmented financial support, limited funding capacity, not freeing all PHC services, fear of deportation, and delayed referral.

Even though it is emphasized in the Sixth Five-Year Development Plan that all foreigners living in Iran should be covered by health insurance [33], UIs still lack insurance coverage [34]. Therefore, they are forced to pay all costs OOP when receiving health care services, which can lead to significant financial hardships for them, and in many cases, they refuse to request needed services and follow their treatment. Immigration status, indeed, has a crucial role in a lack of insurance coverage, as demonstrated by Vargas-Bustamante et al. (2014) in the USA, where UIs face many difficulties in getting such insurance coverage [35]. Also, a qualitative study by Campbell and colleagues revealed that UIs in Canada were not able to obtain medication because of high OOP [36]. In this regard, in Malaysia, UIs have been excluded from social security and health policies that could confront this vulnerable group with catastrophic health expenditures (CHEs) [37]. Meanwhile, by establishing a health insurance program for immigrants, the Thai government has made it possible for even UIs to participate in this program, which is supported by the Ministry of Health [38]. Although UIs are excluded from public health insurance programs in many areas, studies have shown that their insurance coverage can be very effective in saving costs [39, 40].

Besides the aforementioned issues, UIs’ failure to seek health services on time will lead to a significant financial burden on the health system. Various factors, such as the fear of deportation and high health costs, prevent UIs from visiting medical centers as much as possible. The concern about the late referral of UIs with severe health conditions has been indicated in other studies in different care settings [41–43]. This late referral can noticeably increase the cost imposed on the health system by increasing the severity of the disease and physical problems. In this regard, a recent cohort study demonstrated how scheduled vs. emergency dialysis for UIs with end-stage renal disease can significantly improve health outcomes and reduce health care costs [44]. Furthermore, another study conducted in California has indicated that by allowing UIs to participate in insurance schemes and having access to low-cost preventive interventions, we can curb the resource strains on health care providers [45]. Therefore, it is necessary for governments, especially those of developing countries such as Iran, which have limited financial resources, to provide a financing platform for UIs to benefit from health services, especially preventive interventions. In fact, such a strategy has the potential to significantly reduce the financial burden on the health-care system in the medium and long term.

It is undeniable to say that the economic conditions of the host country are also very important in providing services for this group of immigrants [46–48]. Iran’s economy has faced many challenges in recent decades, which have become more complicated after the imposition of international sanctions [49–51]. Under such conditions, the financial resources of Iran’s health system have faced serious limitations, which makes it very difficult to allocate financial resources for the health services of UIs. Following the 2008 financial crisis, such conditions can be found in some high-income European countries [46–48]. Some of the southern coastal countries of Europe (Italy, Spain, and Greece) [48, 52], which are also the main entry points for immigrants, adopted strict austerity measures in order to deal with the economic challenges that arose. Because health-care coverage was reduced and user fees were raised, these policies had a significant impact on both citizens’ and immigrants’ access to health-care services [46, 48, 52]. Besides this, fragmentation of health financing is another serious challenge of Iran’s health system, which has caused resource pooling and risk sharing to not take place well [53, 54], in addition to leading to a lack of transparency, inefficiency, inequity, and corruption [55, 56]. Such a situation has also affected the financing of health services needed by UIs. Similar circumstances can be found in Germany’s health-care system, which has a high level of fragmentation and decentralization in health-care financing for refugees and asylum seekers [57–59]. Based on the evidence, it is necessary to integrate the financing of minority populations like refugees and immigrants into the current health financing mechanisms to have better revenue collection, resource pooling, and purchasing [57, 60]. Generally, despite the fact that there are several fragmented programs to support the UIs financially to access their needed health services [61, 62], as the organized financial protection mechanism is not defined for them, they have struggled with financial barriers to utilizing services.

In addition to the many challenges raised in this study, policy solutions were also proposed to improve the financing of the services needed by UIs, of which the main one is the formation of a health insurance program for UIs within the current Iranian health insurance mechanism. As an incentive, having health insurance can be a requirement for UIs to be allowed to stay in the country legally. This financing strategy can, indeed, significantly reduce management costs and, at the same time, facilitate risk sharing among insured people [60]. This is because both models of financing health services, including the consideration of foreign groups such as refugees in the current insurance schemes and the consideration of a separate ring-fenced fund, are seen among European countries, but still, due to the lack of sufficient evidence, it is not possible to say whether each is more effective [63]. However, it has been demonstrated that by adapting the ex-ante financing approach, we can minimize the problems associated with pooling and distributing resources while having more equity in this process [13, 53, 60]. Furthermore, it should be noted that insurance coverage should be mandatory for all immigrants. Because scientific evidence shows that whenever participation in health insurance plans was optional, adverse selection occurred and insurance mechanisms such as risk sharing did not work well [64, 65]. Therefore, moving toward a mandatory insurance mechanism can significantly improve the financial protection for all immigrants, including UIs.

During this study, several innovative solutions were proposed in order to provide financial resources for the health insurance fund for UIs in Iran. First, many Afghans in Iran have good financial capacities that can be used as one of the revenue sources to finance UI health services. Considering that almost all UIs in Iran are Afghan, the willingness of their countrymen to participate in such an insurance program is likely to be higher. But it should be said that even though community ties could be a reason for legal Afghan residents to give, this policy would be unfair to legal Iranian residents in terms of their rights and responsibilities, and it would be hard and expensive to put into place. In addition, by adopting such an approach, non-Afghan UIs would remain uninsured. Therefore, it may be possible to strengthen the voluntary participation of wealthy immigrants to provide financial resources by creating non-governmental and charitable institutions, which is both fairer and more efficient [66, 67]. Further, earmarking a proportion of taxes paid by immigrants could be another revenue source for health insurance for UIs. This revenue source, if collected well, can actually facilitate the equitable and sustainable financing of health services for this group of immigrants [37]. However, we have to pay attention to the fact that due to the defects in Iran’s tax system and the use of different approaches by immigrants to evade taxes, such a financial source may not be very achievable. Nonetheless, if there are significant tax revenues from immigrants, a part of it can be earmarked for financing their health services to avoid deviation in the allocation and distribution process [55, 60]. Receiving monthly insurance premiums from immigrants is another suggested solution; however, considering that many immigrants have large families [34, 68] and that the insurance premium rate is calculated based on the number of family members, they are not very willing to participate in insurance plans. On the other hand, due to the lack of transparency regarding the income and assets of immigrants, it is not possible to determine the insurance premium based on these indicators. In response, it is essential to develop an effective registration system for immigrants in Iran that provides the possibility to accurately check the number of family members, the amount of income, and the amount of their assets in order to apply a means-testing system to estimate the insurance premium for them.

Participatory governance has been recommended as a requirement for organized financial protection for UIs in Iran, as previous studies indicated that the optimal health financial protection model in Iran follows the shared-governance pattern [69], which requires that a council consisting of all the actors and stakeholders with clear responsibilities and duties make the decisions, and so network governance is an ideal model for the governance of health financing for UIs in Iran. Although the creation of such governance models with the participation of various stakeholders can provide a platform for fair financing and providing quality services for different groups of immigrants in Iran, considering the conditions governing Iran’s health and social systems, such a strategy will be very time-consuming and costly. In response, it is necessary for charitable and non-governmental institutions to play a more prominent role in facilitating financing and providing health services for UIs in the country. Indeed, the establishment of audit and surveillance structures by these institutions can guarantee the quality of services provided while increasing the efficiency of the financing process. For example, UNHCR assigned a third-party auditor aiming to ensure a desirable level and quality of care for Syrian refugees in Lebanon [70].

Notably, it has been suggested that, in order to have a larger pool of financial resources and also to reduce the financial burden on the host country, insurance programs at the supranational level could be considered [13, 57]. Since Iran is located in a region where many rich countries are present, it is possible to increase the obligations of regional countries towards this group of people by forming regional health insurance for immigrants (both documented and undocumented) while transferring the financial risk from Iran. In this regard, some regional organizations like the Organization of Islamic Cooperation (with a membership of 57 Islamic countries), the Shanghai Cooperation Organization, the Economic Cooperation Organization (ECO), and the D-8 Organization for Economic Cooperation (including eight developing Islamic countries) have the capacity to establish such a health financing mechanism for immigrants. In addition, it is possible to use the capacity of rich western countries as well as international organizations to provide financial resources for such health insurance funds [67, 71, 72]. This requires the use of the principles of health diplomacy at different global and regional levels in order to inform the representatives of the countries during the summits about the importance of the health of populations such as immigrants and gain their support to participate in financing [73–75].

### Limitations

Despite its strengths, the present study also faced several limitations. First, UIs did not participate in this study, and the findings are devoid of their experiences. As a result, it is suggested that in future studies, the experiences of UIs be investigated. Second, the current qualitative study has only identified the challenges of financing services for UIs without calculating the amount of OOP payments, the rate of CHE, or impoverishment expenses for them. Therefore, future quantitative studies may estimate these indicators for UI in Iran.

## Conclusion

A number of policy solutions have been recommended in this study to mitigate the financial hardships when UIs use health care services, such as creating insurance coverage for UIs, which can be funded by using the capacity of Afghans with adequate financial resources to support the UIs, earmarking a proportion of taxes paid by immigrants, and utilizing the resources of international organizations such as the UNHCR, with some potential facilitator tools such as receiving insurance premiums from immigrants on a monthly basis. Nonetheless, while it is proposed to charge UI premiums, they must be lower than for other groups in order to encourage them to participate more in the insurance scheme. Notably, a considerable proportion of identified solutions are related to strengthening the governance of health care financing for UIs, including taking advantage of the capacity of international and regional organizations, developing a national health map for UIs, moving towards ex-ante financing approaches, and formulating policies through a network governance model. To progress on the path to UHC, there is no way but to include the UIs and ensure their access to health services in host countries, but in the case of a developing country such as Iran that already has some financial constraints in health financing, it needs more international support and attention. Indeed, in order to reduce the financial pressure on developing countries such as Iran, which have a significant population of immigrants, it is necessary to strengthen the role of developed and rich regional and international countries in financing health services for immigrants.

## Data Availability

The datasets used and/or analysed during the current study are available from the corresponding author on reasonable request.
